# Flow Chamber System for the Statistical Evaluation of Bacterial Colonization on Materials

**DOI:** 10.3390/ma9090770

**Published:** 2016-09-10

**Authors:** Friederike Menzel, Bianca Conradi, Karsten Rodenacker, Anna A. Gorbushina, Karin Schwibbert

**Affiliations:** 1 Department 4, Materials and the Environment, Bundesanstalt für Materialforschung und -prüfung (BAM), Berlin 12205, Germany; Friederike.Menzel@bam.de (F.M.); bianca.conradi@web.de (B.C.); Anna.Gorbushina@bam.de (A.A.G.); 2 Helmholtz Zentrum München, Institute of Computational Biology, Neuherberg 85764, Germany; karsten@rodenacker.de; 3 Department of Biology, Chemistry and Pharmacy, Freie Universität Berlin, Berlin 14195, Germany; 4 Department of Earth Sciences, Freie Universität Berlin, Berlin 14195, Germany

**Keywords:** subaerial and subaquatic biofilm, *Escherichia coli*, image analysis, microscopy, biofilm reactor, biofouling

## Abstract

Biofilm formation on materials leads to high costs in industrial processes, as well as in medical applications. This fact has stimulated interest in the development of new materials with improved surfaces to reduce bacterial colonization. Standardized tests relying on statistical evidence are indispensable to evaluate the quality and safety of these new materials. We describe here a flow chamber system for biofilm cultivation under controlled conditions with a total capacity for testing up to 32 samples in parallel. In order to quantify the surface colonization, bacterial cells were DAPI (4`,6-diamidino-2-phenylindole)-stained and examined with epifluorescence microscopy. More than 100 images of each sample were automatically taken and the surface coverage was estimated using the free open source software g’mic, followed by a precise statistical evaluation. Overview images of all gathered pictures were generated to dissect the colonization characteristics of the selected model organism *Escherichia coli* W3310 on different materials (glass and implant steel). With our approach, differences in bacterial colonization on different materials can be quantified in a statistically validated manner. This reliable test procedure will support the design of improved materials for medical, industrial, and environmental (subaquatic or subaerial) applications.

## 1. Introduction

Prevention of microbial contamination and resulting biofilm formation on surfaces is one of the biggest challenges in biomedical and industrial settings. The use of indwelling medical devices such as pacemakers, prosthetic joints, and venous- or urinary catheters poses risks of bacterial infections. In fact, 95% of urinary tract infections are attributed to the usage of urinary catheters, and 87% of bloodstream infections are associated with intravascular devices [1]. Microbial contamination is also problematic in a large number of industrial processes and causes high costs for cleaning and maintenance [2]. For instance, the overall costs associated with hull fouling on a mid-sized vessel were estimated to be $56 M per year [3]. The development of biofilms takes place as a result of a series of events: microbial surface attachment, cell proliferation plus the formation of microcolonies, and the production of extracellular polymers [4,5]. Among these, bacterial adhesion is a key step in biofilm formation and numerous factors like surface charge, roughness, hydrophobicity, and predisposition to protein adsorption influence the initial colonization of bacteria on a material surface [6,7,8]. Therefore, new materials with low susceptibility to bacterial adhesion are in high demand [9,10,11,12]. 

To assess and compare the antimicrobial efficacy of new materials and their ability to repel or attract bacterial cells, the choice of the adequate test method is critical. Standardized methods currently in use are designed to test the ability of plastics, metals, ceramics, and other non-porous surfaces to inhibit the growth of bacteria or even kill them. ASTM E2149 (Standard Test Method for Determining the Antimicrobial Activity of Immobilized Antimicrobial Agents under Dynamic Contact Conditions) is often used to measure the antimicrobial activity of non-leaching, irregularly shaped, or hydrophobic surfaces. If an antimicrobial object can be tested as a flat coupon, the Japanese Test for Antimicrobial Activity and Efficacy (JIS Z 2801, ISO 22196 Measurement of Antibacterial Activity on Plastic Surfaces) is usually recommended. Although these methods have emerged as industry standards, they only focus on the survival of bacteria in solution, which contains the material under investigation. However, they do not assess bacterial adhesion to the surface. Knowing the exact number of bacteria attached to a surface is important information in order to evaluate new materials concerning their ability to reduce the formation of biofilms. 

Many analytical techniques to study and quantify microbial colonization on abiotic surfaces are presently in use [13,14]. Depending on the kind of material, its mode of action and possible application site, different methods and experimental setups for investigation of the susceptibility to bacterial colonization might be required. Regardless of the method or combination of methods chosen, the first step for comparative testing is the reproducible, standardized colonization of materials.

Herein, we report on a flow chamber system for biofilm cultivation that combines easy handling with a statistically safeguarded, uniform distribution of bacterial cells on material coupons, irrespective of the coupon position in the chamber. The chamber can be run under standardized conditions and it proves particularly versatile for material samples of different sizes and shapes. Following colonization, the samples can be analyzed by microscopy or according to ASTM E2149 or JIS Z 2801/ISO 22196 by indirect methods like removal of adhered bacteria and determination of the colony forming units. Furthermore, we present an image analysis procedure based on the free open source software g’mic (http://gmic.eu), which allows for a statistically validated easy assessment of the surface coverage by bacteria. 

## 2. Results

### 2.1. Design of the Flow Chamber System

**Figure 1:**
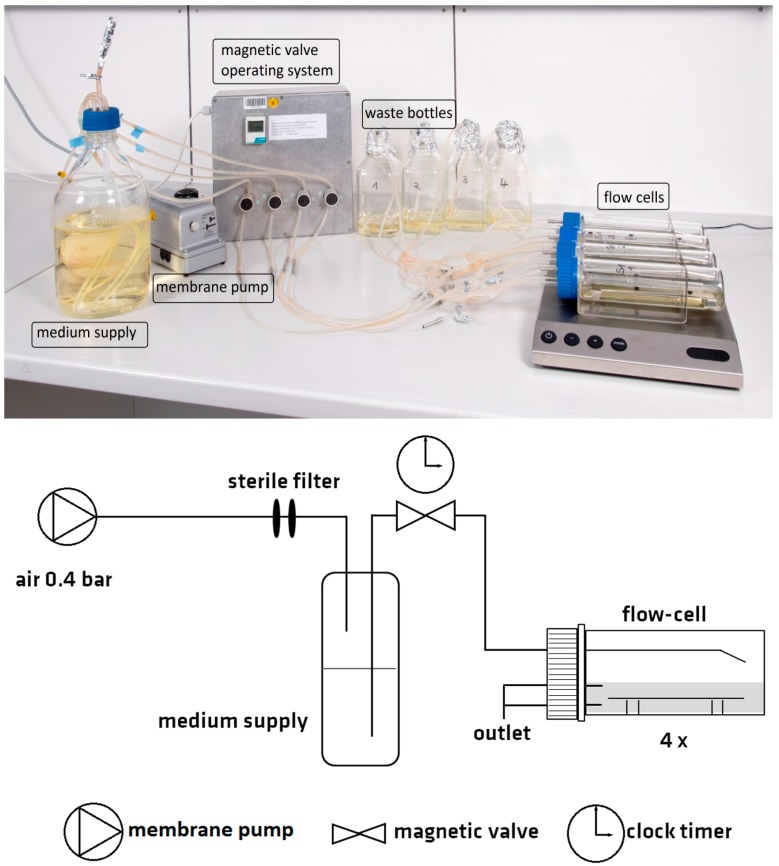
Experimental setup of the flow chamber system. A membrane pump generates an air pressure in the medium supply container and fresh medium is supplied by controlled opening and closing of the magnetic valves. A flow rate of 16 mL·h^−1^ (open valves for 3 s every 15 min) was applied in the described experiments.

The main constituents of the flow chamber system presented in this study were four cylindrical glass vessels (200 mm length, 50 mm in diameter), each equipped with a stainless steel tray with the dimensions of 153 × 29 mm^2^ (total area: 44.4 cm^2^) to serve as the specimen holder (Figure 1). The tray has a capacity for eight coupons 37 × 14 × 1 mm^3^ in size, but could be loaded with specimens of different dimensions and geometrical shapes with a maximum height of 5 mm. The flow chambers, which could be used individually or in parallel, were connected with silicon tubes to medium supply and waste containers. To assure an equal distribution of fresh medium to all chambers used in parallel, a combination of a membrane pump and magnetic valve system was chosen. The air pressure was generated in the container for medium supply by the membrane pump. At defined times, the valve system opened and allowed fresh medium to flow into the chambers. Frequency and duration of medium injection was selected by the user. At the same time, used medium was drained into the waste containers. 

**Figure A1:**
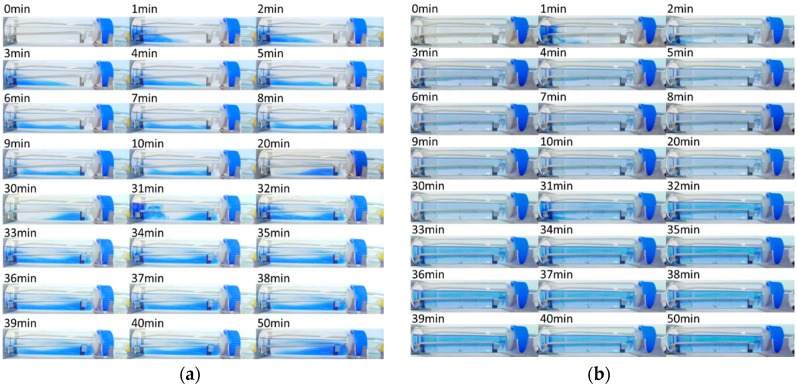
Distribution of Remazol in the flow chamber, injection in the beginning and after 30 min for 6 s (16 mL·h^−1^). (**a**) Without stirring; (**b**) with a stirrer set to 300 rpm.

**Figure A2:**
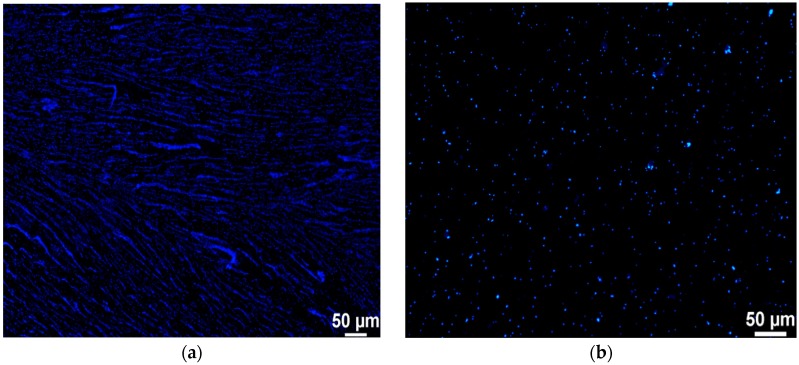
*Staphylococcus pseudintermedius* stained with 4`,6-diamidino-2-phenylindole (DAPI): Cultivation with a medium flow of (**a**) 21 mL·h^−1^ (10.5 mL every 30 min) and (**b**) 16 mL·h^−1^ (4 mL every 15 min).

The rapid and homogenous distribution of injected fresh medium was verified with dye experiments (Appendix A, Figure A1). Different combinations of pulse frequency, pulse length, and stirrer rotation were tested. These experiments revealed that a stirrer at the bottom of the chamber was necessary to assure homogenous distribution approximately four min after medium injection. We chose a slow flow rate of 16 mL·h^−1^ (open valves for 3 s every 15 min), because a higher flow rate impeded an even distribution of bacterial colonization on the material surface and resulted in a wave-like colonization pattern (Appendix A, Figure A2).

Mono-cell-layer adhesion was rated as bacterial colonization and assessed by epifluorescence microscopy, which is more distributed in laboratories than a confocal microscopy. Pretests showed that nutrient reduced mineral salt medium fulfilled the requirement for bacterial colonization and a diluted (1:25) mineral salt medium MM63 [15] was used for the colonization experiments.

**Figure B1:**
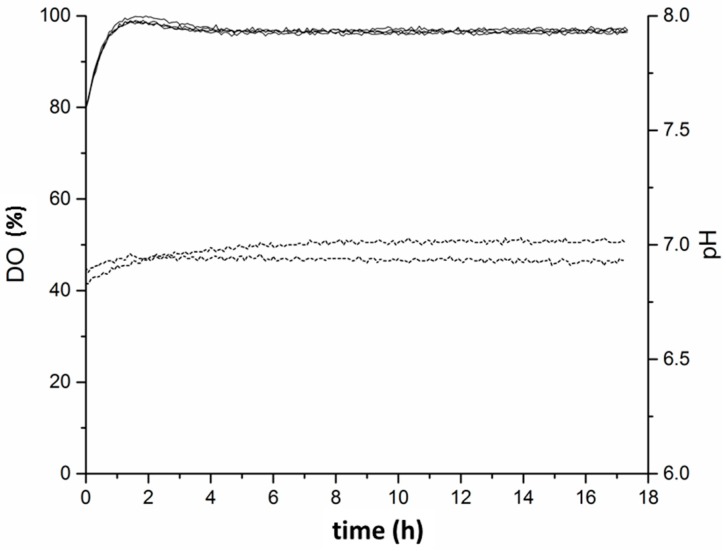
Dissolved oxygen (DO) and pH measurement of one flow chamber system experiment with glass coupons. Straight lines: DO results of two chambers, each with two measuring points; dashed lines: pH measurement in two flow chambers, each with one measurement point.

The dissolved oxygen content (DO) and pH of the cultivation media were measured throughout the whole experiment. The dissolved oxygen level did not fall below 80% during the whole incubation time, which demonstrated that oxygen supply was sufficient under the applied conditions. The mineral salt medium was buffered and only little changes in the pH value (<0.4) occurred (Appendix B, Figure B1).

The viability of the *E. coli* cells in the cultivation chamber was checked and has proved to be stable at end of the experiment (2.1–4.1 × 10^3^ CFU·mL^−1^).

### 2.2. Quantification of the Initial Bacterial Adhesion

On the basis of the free open source software g’mic, we developed a semi-automated analysis tool for the quantification of bacterial colonization on material surfaces. The script and a flow chart of the processing steps (Figure S1) are provided in the Supplementary Materials and thus this analysis tool is available for usage and adaptation according to demand. This semi-automated analysis procedure allows for a fast and accurate analysis of a high amount of coupons.

**Figure 2:**
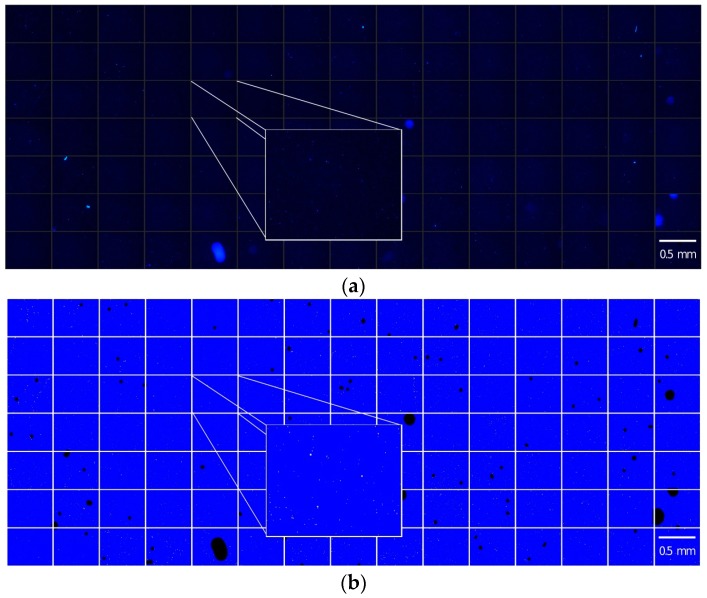

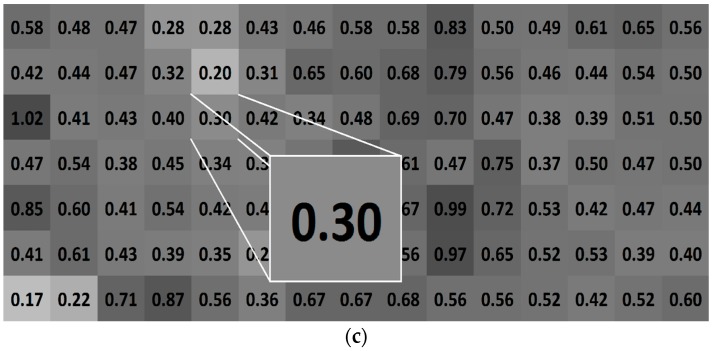
Exemplary procedure for the estimation of bacterial coverage on a glass surface (**a**) Aligned microscopic images (105); (**b**) Overview image including the results of segmentation; (**c**) Calculated bacterial surface coverage for each image.

For an easy and quick assessment, overview images were generated from each coupon sample to elucidate the colonization characteristics of the material under investigation (Figure 2a). 

At first, segmentation of the cells from the background was done by a threshold which was automatically calculated by the triangle algorithm [16]. In short, this algorithm draws a line between the highest and the lowest signal of the pixel intensities detected in the histogram. The distance between this line and the histogram is calculated for every point, and the threshold is set at the point where the distance is at its maximum. This algorithm was chosen because the histogram of the pixel intensities showed a unimodal distribution. Segmentation was done for each image separately. Detected objects, which were smaller than six pixels (1.4 μm^2^) or larger than 400 pixels (94.48 μm^2^), were considered as artefacts and removed automatically. Afterwards, overview images were generated that included the results of the segmentation and the original pixels for one certain coupon (Figure 2b). These overview images allowed for a quick evaluation of image quality and segmentation results and provided the possibility to recognize and remove blurred images and false detected objects quick and easily. 

The bacterial surface coverage, given in percent, was estimated after thresholding. The area of segmented bacteria (A) and the reference area (Aref) were measured. The surface coverage (A/Aref) on a certain material coupon was calculated for every image and for the total coupon. Results were given in percent (A/Aref * 100) (Figure 2c).

### 2.3. Determination of the Minimum Image Number

**Figure 3:**
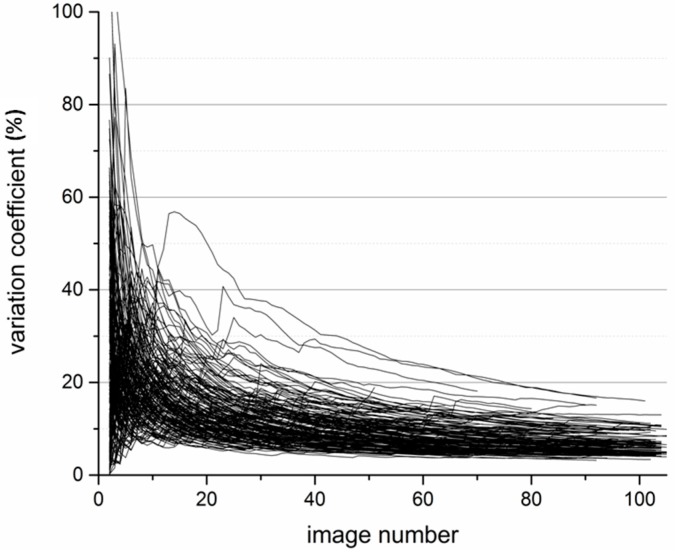
Decrease of the variation coefficient in dependence of the image number.

To address the question of how many pictures are necessary to reach statistically rigorous results, the cumulative variation coefficient was calculated for each coupon of the experiments. One hundred and five images were necessary to lower the coefficient to 13% or below (Figure 3); for a variation coefficient below 20% at least 75 images were necessary.

### 2.4. Assessment of Bacterial Adhesion in Dependence of the Coupon Position in the Flow Chamber System

In order to compare the ability of different materials to attract bacterial cells, it has to be ensured that bacterial colonization is independent of the coupon position in the flow chamber system. 

**Figure 4:**
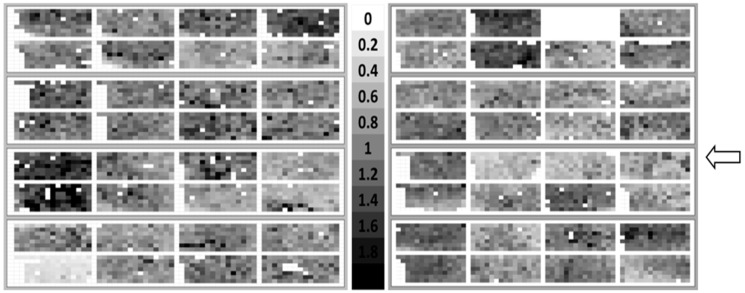
Fingerprints of colonization pattern created according to the data processing procedure summarized in Figure 2. Two independent trials with 32 and 31 implant steel coupons are shown. Each trial was performed with four flow chambers, each equipped with eight coupons. Numbers indicate bacterial coverage in percent, blank areas represent invalid images due to blurring and false detected objects and were not included in the evaluation. The arrow indicates the position of the medium injection site.

Therefore, a high number (105) of images for each coupon were captured to analyze a large area of the coupons. This number of images proved to be just sufficient enough to generate fingerprints of each coupon, with each fingerprint depicting the colonization pattern of one coupon. These fingerprints revealed no dependency of the bacterial colonization either on the position of the material coupon or on the flow direction in the chamber (Figure 4). There were differences between the coupons, but these differences were randomly distributed (no obvious variation).

### 2.5. Reproducibility of Results

**Figure 5:**
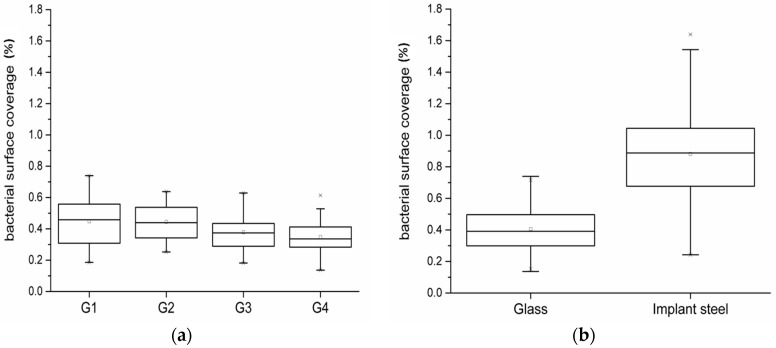
Box-Whisker plot for the results of (**a**) the four experiments with glass (each box includes the bacterial coverage data (%) of up to 32 material coupons, for a total of 107); and (**b**) for the experiments with glass (G1–G4) and implant steel (63 coupons, results shown in detail in Figure 4).

To assess the reproducibility of the results obtained with the flow chamber system, colonization experiments with glass coupons were carried out. Since there was no specific pattern recognized on the coupons, the surface coverage (%) of the whole coupon was calculated for the analysis of differences between glass and implant steel. The results for each flow chamber system experiment (each with four flow chambers, each flow chamber equipped with up to eight coupons) were taken together and checked with the Shapiro-Wilks-, the Kolmogorov-Smirnov-, the Lilliefors-, and the Anderson-Darling test for normal distribution. All data sets were normally distributed (*α* = 0.05). Afterwards, one way analysis of variance (ANOVA) was performed with these data sets. No significant differences were observed between the four trials with glass coupons, when the significance level was set to 0.1%. When the significance level was set to a lower level, significant differences between the first and the last glass trial were recognized, but nevertheless the differences between the mean values were less than 0.1% (Figure 5a). Furthermore, the confidence intervals of the four trials were overlapping, which underlines that the results were highly reproducible.

Finally, the data sets for the bacterial surface coverage for glass and implant steel coupons were compared (Figure 5b). The surface coverage was almost doubled on the implant steel in comparison to the glass material. Both data sets were normally distributed (*α* = 0.05). The t-test revealed significant differences between glass and implant steel coupons (*α* = 0.001).

## 3. Discussion

In this study, we present a flow chamber system designed for the testing of materials regarding their susceptibility to bacterial adherence. This test system combines convenient handling with great flexibility concerning shape and size of the material specimens. Microbial strains, and medium and culture conditions can be selected according to the user’s demand. 

Two general approaches for bioreactors suitable for biofilm testing are known from literature. In the classical flow chamber systems, or “plug flow reactors”, the specimens are exposed to bacterial cultures in a chamber with an inlet and an outlet on opposite sides. Typical examples are drip flow reactors, classical flow chambers, and modified Robbins devices. All these systems have in common that the turbulence is low, depending on the flow velocity. However, conditions inside these chambers are not constant and change progressively throughout the reactor [17,18]. Mixing occurs only by diffusion, which we had considered as the main drawback of these systems. In the second approach, specimens are placed in common bioreactors, ensuring constant conditions in the whole liquid phase by mixing with a stirrer. Typical examples are the CDC (Centers for Disease Control), the annular, and the rotating disc biofilm reactor [17,18]. The use of a stirrer enhances shear forces and turbulences in the liquid phase, which can influence the adhesion behavior of bacteria [19,20,21]. On the other hand, biofilm formation can be reduced under static compared to dynamic conditions [19,21].

We combined the advantages of both systems. The addition of fresh medium was performed regularly to allow limited growth in the flow cells. Medium inlet and outlet are located at opposite sides of the bioreactor. Constant conditions were ensured by mixing with a stirrer centered on the bottom of the flow chamber. Moreover, the specimens were located on a tray above the stirrer to reduce the influence of shear forces. The presence of two outlets offers the opportunity to drain the medium periodically. This is a valuable tool to simulate and analyze subaquatic and also subaerial biofilms which are frequently encountered in nature. 

This specific design of the flow chamber system allows (i) homogenous medium supply; (ii) application-related biofilm cultivation; (iii) investigation of specimen with different geometries; and (iv) analysis of a statistically relevant number of samples. 

We decided to use a diluted minimal medium and bacteria which were not in the exponential stage of growth for adhesion experiments. In nature, exponential growth is rather an exception and bacteria have to cope with a reduced supply of energy. Under these conditions, the establishment of a biofilm is promoted because it offers a fitness advantage [22,23]. Furthermore, it is reported that biofilm formation increased in diluted growth medium [24,25] and was described as faster when the growth rate of planktonic bacteria was lower [25]. 

Although biofilm formation is a big challenge in medical and industrial applications, an easy and fast direct quantification method which allows for a reproducible and statistically reliable analysis is still missing [13,26]. Quantification of bacteria directly on the surface reveals more information compared to the indirect methods currently in use. Therefore, we developed a direct method based on a semi-automated analysis for rapid discrimination of materials concerning their susceptibility for bacterial adhesion. The generation and pre-analysis of overview images also facilitate the evaluation of the materials.

A commonly used indirect method is the dislodgement of bacterial cells from the specimen by sonication, followed by plate spreading and colony counting (colony forming units = CFU). The detachment procedure is the crucial step during analysis and the application of different cell detachment procedures hamper the comparability of the results [13,27]. A weak detachment procedure does not ensure a complete detachment of the cells [13], whereas an inappropriate power density of the sonication regime leads to the thinning of the cell membrane, which decreases the number of detected colonies [28]. Furthermore, different sonication times are applied, eventually resulting in different CFU numbers [27,29,30,31]. Moreover, when the detached bacterial cells appear in aggregates, the detected CFU will be too low [13]. Cell count determination by microscopic analysis directly on the material surface usually results in higher values compared to the results obtained by sonication and determination of CFU [32]. Besides, CFU methods are labor-intensive, time-consuming, and have a high material throughput [18]. Colorimetric and fluorescent methods are faster and more convenient [33], but nevertheless, most colorimetric methods are done in microtiter plates, which are not suitable for testing bacterial colonization on different surfaces with different geometries. With indirect methods only the total number of adhered bacterial cells is detectable and it is not possible to focus on weak points of a certain specimen like threads or grooves on screws or stents, for example, or the interface of two combined materials. 

Various methods for the quantification of bacterial colonizations are known from literature and a “gold standard” does not exist, since each method will give an answer to a specific question [14]. The specific application of the material has to be considered when choosing the right cultivation and quantification method [18,21]. Nevertheless, the choice of the biofilm cultivation method (static or flow conditions) seemed to be critical for adhesion behavior, and there is obviously a need for standardized methods and agreed protocols if different material surfaces will be compared. The developed flow chamber combined with the quantification step is ideally suited for this purpose.

Variations were recognized within a certain material specimen, which can be caused by material inhomogeneities or biological fluctuations. However, we showed that with a high number of images per material specimens statistically rigorous results were possible. The variation coefficients fell below 13%, which underlined the importance of a high number of images to gain reliable statements about material differences. We present here a method with which already small changes between materials can be detected, because of its high statistical resolution.

In sum, we developed a flow system which is cost efficient and easy to handle. It can be used for material specimens of various sizes and is not only appropriate for the testing of different materials and coatings, which are usually provided as flat coupons, but also for the testing of specimens such as whole implant devices like screws, fixators, or dental prostheses. 

## 4. Materials and Methods

### 4.1. Bacterial Strain and Growth Media

*Escherichia coli* W3110 (kindly provided by Prof. R. Hengge, Freie Universität Berlin (now Humboldt-Universität zu Berlin), Berlin, Germany) was used for the experiments. A colony was picked from an agar-plate (MOPS, 0.033 M KH_2_PO_4_, 0.25 M Glucose, 20 g·L^−1^ Agar) and cultivated overnight in the minimal medium MM63 [15]. After reaching the stationary phase, the culture was diluted to OD_540_ = 1 with fresh medium and used for inoculation of the flow chamber system.

### 4.2. Test Coupons

Glass material was purchased from Marienfeld, Lauda-Königshofen, Germany and cut into the appropriate size of 37 × 1 × 1 mm^3^ by hand. Coupons made of implant steel, also 37 × 13 × 1 mm^3^, were purchased from Königsee Implantate GmbH, Allendorf, Germany. Coupons were washed in detergent solution, disinfected for 20 min in 70% ethanol solution, air dried, and transferred aseptically to the flow chambers.

### 4.3. Operation of the Flow Chamber System

Cylindrical glass vessels (20 cm, 5 cm in diameter, Ochs, Bovenden/Lenglern, Germany) were used as biofilm reactors. The reactors were closed by silicone rubber plugs equipped with one inlet (V4A steel, diameter: 3.5 mm) and two outlets (both glass, diameter: 3 mm) Figure 1. The upper outlet limited the maximum fluid level to approximately 140 mL, the lower one was used for draining the reactor. The components of the flow chamber system (medium supply, bioreactor, and waste) were connected via silicon tubes. Silicone rubber plugs (already equipped with inlet and outlets), the specimen holders, and the tubing plus electronically controlled magnetic valves needed for medium distribution were purchased from Durakult, Berlin, Germany. Distribution of fresh medium to the individual flow chambers was achieved by a combination of air pressure driven medium flow and magnetic valve systems as described in Section 2.1. Continuous mixing of the medium was achieved by a magnetic stirrer (10 × 3 mm^2^) centered at the bottom of the flow chamber. The system was run at 37 ± 2 °C in an incubator (Innova 44 Lab Shaker Series, New Brunswick Scientific’s, Nürtingen, Germany). 

### 4.4. Bacterial Attachment Tests

Pretests had shown that the usage of diluted MM63-medium (4% v/v) with a medium exchange rate of 270% per day, and an incubation time of 18 ± 1 h resulted in a mono-cell-layer colonization on glass surfaces, which was required for analysis by epifluorescence microscopy. 

Each flow chamber was inoculated with 100 μL of the preculture and flushed with 140 mL 4% MM63-medium. After an initial sedimentation phase of one hour [34,35,36], 4 mL of fresh 4% MM63-medium was injected every 15 min. 

The flow system was stopped after 18 ± 1 h of incubation, flow chambers were emptied, and the material coupons were removed, washed with sterile PBS solution (8.0 g NaCl, 0.2 g KCl, 1.44 g Na_2_HPO_4_, 0.24 g KH_2_PO_4_, pH 7.4), fixed with 3.7% formaldehyde (Sigma-Aldrich, Deisenhofen, Germany) in PBS solution for 2 h at 4 °C, and rinsed afterwards with sterile deionized water. Coupons were dried and stored at room temperature.

To test cell viability, colony forming units were determined in the medium phase when the flow system was stopped. Medium solution was diluted with MM63 medium lacking glucose and plated on STD1-agar-plates (Merck, Darmstadt, Germany), each dilution was plated in triplicate. After incubation at 37 °C for 24 h, colony forming units were counted. The cell viability test was performed in quadruplicate.

### 4.5. Microscopy

Material coupons were stained with 20 μL DAPI-solution (4`,6-diamidino-2-phenylindole, 10 μg/mL, Sigma-Aldrich, Deisenhofen, Germany) for 20 min at 46 °C, washed with deionized water, and dried for 20 min at 46 °C. Afterwards, coupons were covered with 14 μL CitiFluorTM AF87 (Science Services, München, Germany) and examined with an epifluorescence microscope (Ni-U Eclipse, Nikon, Düsseldorf, Germany). For determination of the bacterial coverage (%), an area of 118.6 mm^2^ per material coupon was investigated. Therefore, 105 images (each 0.622 × 0.467 mm^2^, which results in a total area of 30.5 mm^2^) were captured automatically in this area. 

### 4.6. Image Analysis

The free open source software g’mic version 1.6.8 (http://gmic.eu, accessed on 17 December 2015) was used for digital processing and analysis of the microscopic images. Quantification of the initial bacterial adhesion was done as described in Section 2.2. The script is provided in the Supplementary Materials. Fingerprints of the colonization pattern were compiled with Microsoft Excel (2013) using the conditional formatting tool.

### 4.7. Statistical Evaluation

Results were evaluated with OriginPro 9 (OriginLab, Northampton, MA, USA). Normal distribution was checked with four different tests (Shapiro-Wilk-test, Kolmogorov-Smirnov test, Lilliefors test, and Anderson-Darling test). Multiple comparisons of the results were done with one way analysis of variance (ANOVA) and post hoc analysis for pairwise comparison with Tukey’s range test. The significance level was set to 5% for all statistical tests applied (confidence of 95%).

## Supplementary Materials

A short instruction to g’mic (BAM.pdf) and the used script (BAM.gmic) are available online at www.mdpi.com/1996-1944/9/9/770/s1. Both can be used with g’mic version equal to or higher than 1.7.2.

## Abbreviations

ANOVA	analysis of variance
CFU	colony forming units
DO	dissolved oxygen

## Appendix A. Evaluation of the Influence of the Flow Conditions on the Media Distribution within the Flow Chamber and the Colonization Pattern

Remazol Brillant blue R (Sigma Alderich, Milwaukee, WI, USA) was inserted to the flow chamber and the distribution was evaluated with automatically taken pictures. Different medium addition regimes were tested. Figure A1 shows clearly that a stirrer was necessary to ensure a homogenous distribution of the added fresh media (Figure A1).

Additionally, the influence of the flow velocity on the colonization characteristic of *Staphylococcus pseudintermedius* was investigated. Therefore, *S. pseudintermedius* was cultivated in the flow chamber system with complex medium for 26 h, at 37 °C with different flow rates. A flow rate of 21 mL·h^−1^ led to a wave like pattern on the coupon (Figure A2a). When the flow rate was decreased to 16 mL·h^−1^ this pattern was not recognized (Figure A2b).

## Appendix B. DO and pH Measurement

Dissolved oxygen content (DO) and pH of the cultivation media were measured throughout the whole experiment. Therefore, the bioreactor was equipped with non-invasive sensors (PreSens, Regensburg, Germany) for pH and dissolved oxygen (DO). Sterile single use sensor spots for pH measurement and reusable DO spots were glued inside the glass vessel. DO spots were calibrated prior to each experiment according to the manufacturer’s instructions. The dissolved oxygen level did not fall below 80% during the whole incubation time, which demonstrated that oxygen supply was sufficient under the conditions applied. The mineral salt medium was buffered and, therefore, only little changes in the pH value (<0.4) occurred (Figure B1).
