# Supplementary material for: Flow Chamber System for the Statistical Evaluation of Bacterial Colonization on Materials
Source: Materials (Basel). 2016 Sep 10;9(9):770. doi: 10.3390/ma9090770 (PMC5457037; doi:10.3390/ma9090770)
Supplement: Supplementary file 1 [file materials-09-00770-s001.zip › materials-143199-supplementary-1/BAM Sup v2.pdf]

Supplementary Materials: Flow Chamber System for the Statistical Evaluation of Bacterial Colonization on Materials

Friederike Menzel [1,†], Bianca Conradi [1,†], Karsten Rodenacker [2], Anna A. Gorbushina [1,3] and Karin Schwibbert [1,*]

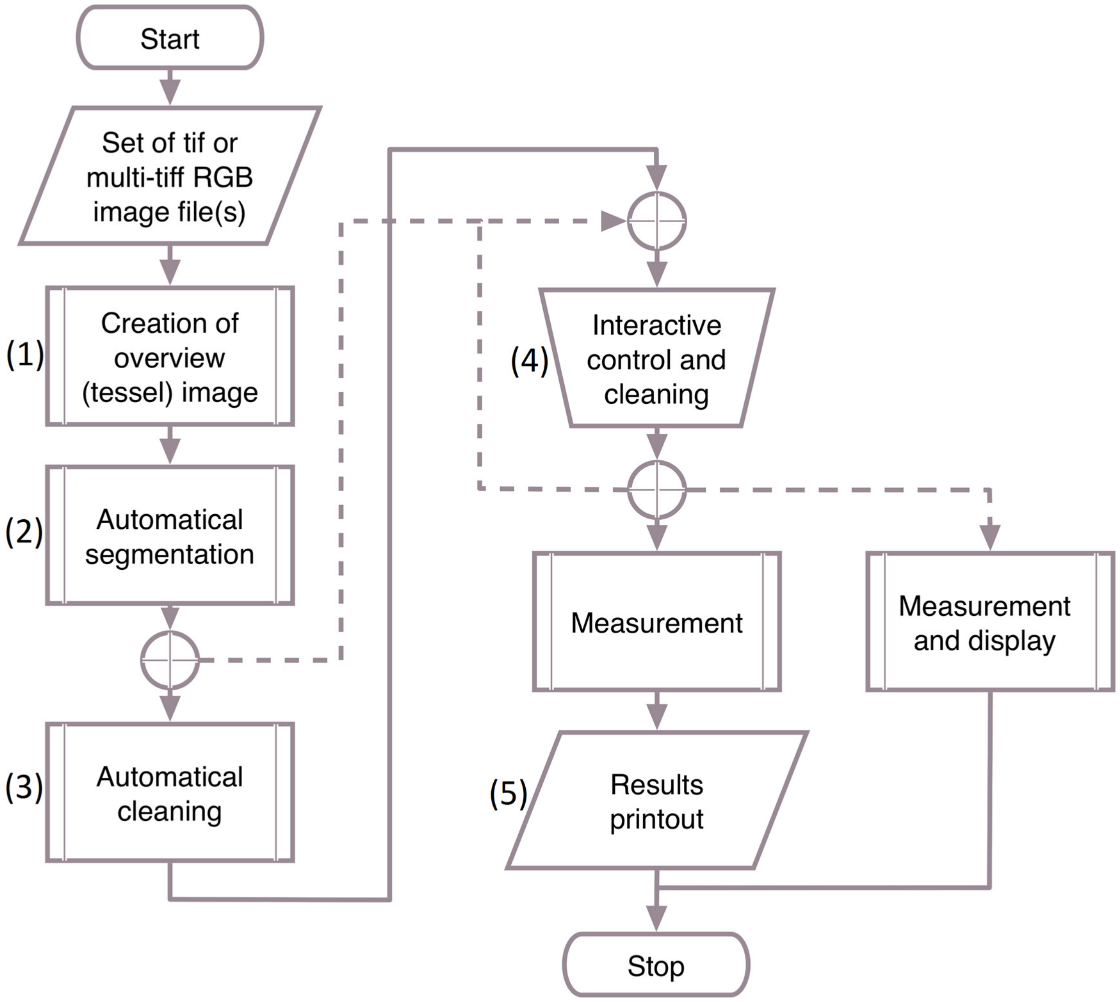

Figure 1. Flowchart of the processing steps, **solid lines** give the standard measuring procedure, **dashed lines** are possible alternatives (without automatically cleaning/repeated interactive control and cleaning/only measurement and display). The commands which were used in each step are presented in Table 1.

The flow chart of the processing steps for the quantification routine is presented in Figure 1. A microscope RGB images with fluorescence signal in the blue channel (B) is required for the analysis, either as a set of tiff images or as one multi-image tiff file. The program gmic has to be installed and the gmic script file BAM.gmic has to be loaded in advance. Afterwards the commands presented in Table 1 can be applied for the quantification procedure.

Table 1. List of applied commands in the quantification procedure. XXX should be replaced with the filenames, which should be identically in each step. In case the image size has a width of 1280 px and height of 960 px, <width>, <height> can be omitted in the commands.

Command Number	Command
(1)	gmic BAMs.gmic xxx.tif -a z -sh 2,2 -s. z -append_tiles[-1-1] 15,7 -rm.. -o ppm:{b}.pgm
(2)	gmic BAMs.gmic xxx.pgm -fh_threshold_p triangle,<width>,<height> -o {b}_triangle.tif,uchar,lzw
(3)	gmic BAMs.gmic xxx_triangle.tif -bc_clean_new 400,15,9,65%,<width>,<height> -o {b}_ok.tif,uchar,lzw[1]
(4)	gmic BAMs.gmic -fh_ue_test xxx.pgm,triangle,,<width>,<height>
(5)	gmic BAMs.gmic -v + xxx_triangle_ok.tif -fh_ue_eval_p <width>,<height>

The segmented image (xxx_triangle_ok.tif) will be cleaned automatically. Objects, which are larger than 400 px and smaller than 9 px are deleted. The area around large objects is enlarged by 15 px before the object is deleted. The whole image will be deleted, if the resulting mask is less than 65 %.

The script file BAMs.gmic defines external commands (e.g. –fh_threshold). For further information, the command: gmic –h <external command name> can be used, to get a short help text for each external command.
